# Measurement of Dynamic Responses from Large Structural Tests by Analyzing Non-Synchronized Videos

**DOI:** 10.3390/s19163520

**Published:** 2019-08-11

**Authors:** Yuan-Sen Yang

**Affiliations:** Department of Civil Engineering, National Taipei University of Technology, 1 Sec 3 Zhongxiao E. Rd. Taipei 10608, Taiwan; ysyang@ntut.edu.tw

**Keywords:** structural experiments, response measurement, camera calibration, signal synchronization

## Abstract

Image analysis techniques have been employed to measure displacements, deformation, crack propagation, and structural health monitoring. With the rapid development and wide application of digital imaging technology, consumer digital cameras are commonly used for making such measurements because of their satisfactory imaging resolution, video recording capability, and relatively low cost. However, three-dimensional dynamic response monitoring and measurement on large-scale structures pose challenges of camera calibration and synchronization to image analysis. Without satisfactory camera position and orientation obtained from calibration and well-synchronized imaging, significant errors would occur in the dynamic responses during image analysis and stereo triangulation. This paper introduces two camera calibration approaches that are suitable for large-scale structural experiments, as well as a synchronization method to estimate the time difference between two cameras and further minimize the error of stereo triangulation. Two structural experiments are used to verify the calibration approaches and the synchronization method to acquire dynamic responses. The results demonstrate the performance and accuracy improvement by using the proposed methods.

## 1. Introduction

Conducting structural dynamic experiments is an important aspect of structural engineering research and the development of structural health monitoring techniques. The quantitative acceleration and displacement responses of the specimens studied in dynamic experiments are used to verify the theory or understanding of materials, devices, or structural systems. Qualitative behaviors such as failure modes, crack patterns, and modal shapes can be used to understand the overall structural characteristics induced by certain types of loadings or ground motions. The measurement techniques for dynamic structural experiments carried out on shake tables are also used for measurement system verification, structural health monitoring, and structural damage identification algorithms.

The experimental data are recorded using various types of local and remote sensing sensors. Local sensors normally have high sampling rates, satisfactory accuracy, and insensitivity to ambient noises. Displacement measurement devices, a type of local sensors, measure the relative movement between two hinges on the device: one fixes on the measured point and the other fixes on a fixed reference such as additional reference frames, which is assumed to be immobile during the measurement. However, as the scale of structural experiments increases, the reference fixities become larger, higher, and easier to deform or vibrate during the experiment, making it difficult to measure satisfactory displacement histories using displacement sensors. In addition, the dramatically increasing number of sensors increases the number of wires and the time required for instrumentation and experimental preparation. All together, these issues increase the difficulties and cost of large-scale shake table experiments.

Another measurement approach is remote sensing exploiting optical tracking systems, light detection and ranging (LiDAR), and image analysis. Some optical tracking systems use passively reflective markers, whose positions are sensed by high-speed infrared or near-infrared cameras [1,2]. These infrared-based optical systems are still expensive, but could achieve an accuracy in the order of 0.01 to 0.1 mm. Image analysis which uses consumer cameras are relatively practical in terms of its low hardware cost. By taking videos or a series of images of structural experiments and employing computer vision techniques, the overall deformation of a specimen can be recorded. In addition, object tracking and image analysis techniques can be used to quantify the movement of certain points on the specimen [3,4], extract object motions [5], structural vibrations in real earthquake events [6], object identification and counting [7], and shape classification [8]. Optical measurement technique based on optical flight time is also used for object distance estimation and 3-D positioning [9] and is further applied for civil applications such as visually impaired aiding [10]. Motion magnification technique to video footages is employed on some shaking table tests to extract the dynamic response of large structures [11,12]. With the employment of three-dimensional computer vision techniques including stereo triangulation and stereo camera calibration handling images from two or more cameras, the three-dimensional displacements of these points can be more accurately calculated. With a dense mesh of points selected as measurement points, the displacement fields [13] and the strain fields [14] of a certain region of the surface of a specimen can be estimated [15]. If surface cracks occur in the region of measurement, accurate displacement fields can be used to estimate the crack patterns [16], as well as quantify crack opening widths [17], crack sliding [18], and crack propagation [19]. Since the images record the overall regions of a specimen, they can be used to measure regional information such as strain fields and crack distribution and development [20], whereas local sensors would require excessive instrumentation and deployment. In addition, the precise positions of regions of interest for measurements can be selected after the experiments [21]. Thus, image analysis has better flexibility and potential for recording the overall information of structural experiments and damage detection [22]. With adoption of the rapidly developing field of machine-learning techniques, image analysis can also be used to detect concrete cracks [23] and pavement cracks [24] by identifying dark lines in images.

Large-scale dynamic structural experiments such as shake table tests bring challenges to image analysis in stereo camera calibration and synchronization [25]. Compared to slow structural experiments subject to monotonic or cyclic loadings, dynamic structural experiments exhibit the effects of inertia forces, viscosity, and material strain rates. Cameras installed inside the specimen would shake during the experiment and require a correction algorithm to partially mitigate the errors [26]. Installing cameras outside the shake table can avoid this shaking, but large-scale measurement of a region makes it difficult to carry out stereo calibration, owing to the lack of large rigid calibration boards. While stereo triangulation is normally used with synchronously controlled high-speed cameras [27], without perfect satisfactory synchronization, the time difference between cameras may cause significant stereo triangulation errors. Some experiments have used a single camera with a v-shaped mirror to create a virtual stereo system to bypass this synchronization problem [28], but this is only suitable for small-scale experiments.

To solve calibration and synchronization problems, this work develops two types of calibration approach: (1) two-stage calibration and (2) single-image calibration, and proposes a synchronization method to reduce the error induced by the cameras’ time difference. Two shake table experiments were used to verify and demonstrate the effects of the approaches and to demonstrate the measurement of dynamic displacements in the experiments.

## 2. Basic Principles and Formula of Image Analysis

The basic image analysis approach employed in this work is based on point measurement. The measurement over a region is based on measurement of multiple points distributed over the region. For each measured point, its three-dimensional coordinates are calculated. The entire time history of a point can be used to further estimate its displacement, velocity, and acceleration, and the time history of multiple points can be used to estimate story drifts, displacement fields, strain fields, and crack patterns and widths.

Calculating the three-dimensional position of a point of interest using image analysis requires a process of coordinate transformation. An arbitrary point in the real world is transformed through five coordinate systems before being projected to an image: world coordinates, camera coordinates, normalized coordinates, distorted coordinates, and image coordinates. Given the extrinsic and intrinsic parameters of a camera, it is feasible to accurately calculate where an arbitrary point in the world would appear in the image (i.e., the image coordinates). The extrinsic parameters include the precise position and orientation of the camera in the world coordinate system. The intrinsic parameters include the focal lengths, the principal point, and the distortion coefficients of the lens of the camera. Several camera calibration methods are widely used to estimate the extrinsic and intrinsic parameters of a camera by taking photos of a known-size regular chessboard [29,30]. Once the extrinsic and intrinsic parameters are known, an inverse transformation from a point in the image (i.e., the image coordinate) to the position of the point in the world (i.e., the world coordinates) can be carried out, except that the depth of the point (i.e., the projection of the distance between the point and the camera to the viewing axis) would be unknown. However, if this point appears in two or more cameras, then the position of the point in the world coordinates can be calculated using stereo triangulation.

**Figure 1:**
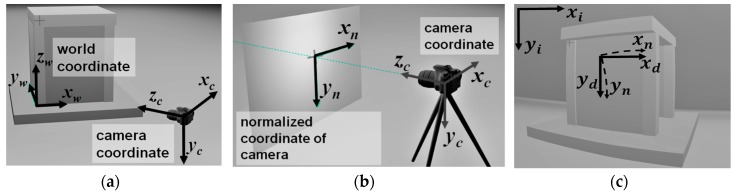
Different coordinate systems used in image analysis. Image measurement involves transformation between different coordinate systems. (**a**) From world coordinates to camera coordinates. (**b**) From camera coordinates to normalized coordinates. (**c**) From normalized coordinates to distorted and image coordinates.

The world coordinates are intuitive coordinates defined by the user. These can be the coordinates used to describe the dimensions of a specimen in a structural experiment, as shown by the three axes *x_w_*, *y_w_*, and *z_w_* in Figure 1a. The world coordinates are normally coordinates that the user is familiar with, because the user may need to manually input the positions of points (i.e., *x*, *y*, and *z* of each point) when calculating the precise position and the orientation of the camera or estimating the geometrical relationship between two cameras. A unit of length in the world coordinates is the physical unit of length defined by the user.

The camera coordinates are a coordinate system defined by the position and orientation of the camera. The *z* axis is the viewing direction of the camera and the *x* and *y* axes are the horizontal (with positive direction to the right) and vertical directions (with positive direction downward) of the camera, respectively (see Figure 1b). There exists a linear transformation between the world coordinates and camera coordinates:

(1) $$\left(\begin{array}{c} \begin{array}{c} x_{c}\\ y_{c}\\ z_{c} \end{array}\\ 1 \end{array}\right)=\left(\begin{array}{cc} \begin{array}{ccc} R_{xx} & R_{xy} & R_{xz}\\ R_{yx} & R_{yy} & R_{yz}\\ R_{zx} & R_{zy} & R_{zz} \end{array} & \begin{array}{c} T_{x}\\ T_{y}\\ T_{z} \end{array}\\ \begin{array}{ccc} 0\;\;\;\; & 0\;\;\;\; & 0\;\; \end{array} & 1 \end{array}\right)\left(\begin{array}{c} \begin{array}{c} x_{w}\\ y_{w}\\ z_{w} \end{array}\\ 1 \end{array}\right)$$

The upper-left 3 × 3 rotational matrix of the 4 × 4 matrix in Equation (1) has only three independent degrees-of-freedom, as it is a normalized orthogonal matrix. The vector ($R_{xx}$, $R_{yx}$, $R_{zx}$)^T^ at the first column of the transformation matrix in Equation (1) is the unit-length vector of the *x* axis of the world coordinates represented in camera coordinates, as are the vectors ($R_{xy}$, $R_{yy}$, $R_{zy}$)^T^ and ($R_{xz}$, $R_{yz}$, $R_{zz}$)^T^. The vector ($T_{x}$, $T_{y}$, $T_{z}$)^T^ is the origin of the world coordinates represented in camera coordinates. These vectors are automatically calculated in the extrinsic calibration procedure, and do not require a user to calculate or measure them manually [29,30].

The normalized coordinate system is a two-dimensional coordinate, which is equivalent to the $z_{c}$ = 1 plane projected from the camera coordinate system. The transformation formula is quite simple:

(2) $$\left(\begin{array}{c} x_{n}\\ y_{n} \end{array}\right)=\left(\begin{array}{c} x_{c}\\ y_{c} \end{array}\right)/z_{c}$$

However, the transformation of a point from the camera coordinates to the normalized coordinates cannot be inverted unless $z_{c}$ is known. Otherwise, this is the only invertible transformation among all coordinate transformations described in this section. Points in the normalized coordinate system are a dimensionless quantity.

The distorted coordinate system describes how an image in normalized coordinates is distorted by the camera lens (see Figure 1c). The lens in a camera has complicated optical behaviors which could depend on the optical design of lens and the manufacturing quality, and these are difficult to describe in a mathematical form without errors. A widely used transformation, reorganized from formula presented in [30] is:

(3) $$\left(\begin{array}{c} x_{d}\\ y_{d} \end{array}\right)=\left(\begin{array}{cc} k+2p_{1}y_{n}+3p_{2}x_{n} & p_{2}y_{n}\\ p_{1}x_{n} & k+2p_{2}x_{n}+3p_{1}y_{n} \end{array}\right)\left(\begin{array}{c} x_{n}\\ y_{n} \end{array}\right)$$

where:

(4) $$k=\frac{1+k_{1}\left(x_{n}^{2}+y_{n}^{2}\right)+k_{2}{\left(x_{n}^{2}+y_{n}^{2}\right)}^{2}+k_{3}{\left(x_{n}^{2}+y_{n}^{2}\right)}^{4}}{1+k_{4}\left(x_{n}^{2}+y_{n}^{2}\right)+k_{5}{\left(x_{n}^{2}+y_{n}^{2}\right)}^{2}+k_{6}{\left(x_{n}^{2}+y_{n}^{2}\right)}^{4}}$$

Note that the relationship between ($x_{n}$, $y_{n}$) and ($x_{d}$, $y_{d}$) in Equation (3) is not linear because $x_{n}$ and $y_{n}$ also appear in the coefficient matrix. The transformation is numerically invertible by using a nonlinear regression solution such as Levenberg-Marquardt method [31].

The image coordinates are used to describe where a point appears in the image. The dimensionless quantity in the distorted coordinates are transformed to a pixel-based image coordinate. The origin of the image coordinates is the upper-left corner of the image. The *x* and *y* axes of the image coordinate are horizontal and vertical, respectively. The transformation between distorted coordinates and image coordinates is as follows; the 3 × 3 matrix is also called the camera matrix:

(5) $$\left(\begin{array}{c} x_{i}\\ y_{i}\\ 1 \end{array}\right)=\left(\begin{array}{ccc} f_{x} & 0 & c_{x}\\ 0 & f_{y} & c_{y}\\ 0 & 0 & 1 \end{array}\right)\left(\begin{array}{c} x_{d}\\ y_{d}\\ 1 \end{array}\right)$$

**Figure 2:**
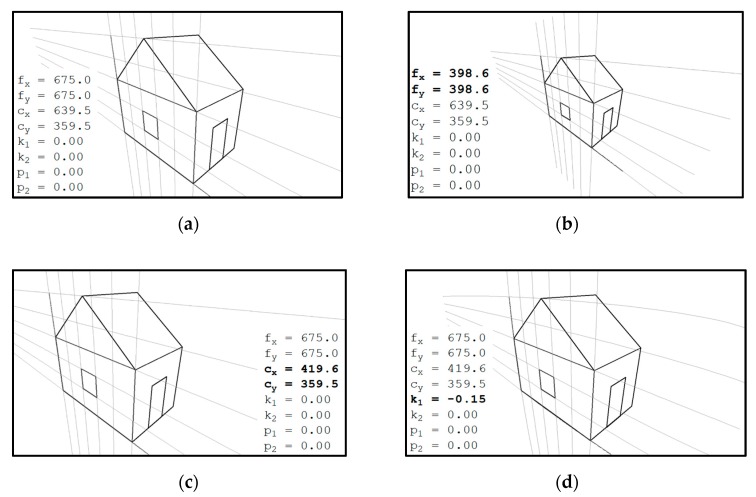
Effects of selected intrinsic parameters. Intrinsic parameters represent the optical properties of a camera. (**a**) house projected in an image. (**b**) Wider angle of view induced by a smaller focal length. (**c**) Translation induced by a biased principal point. (**d**) Distortion induced by a negative coefficient *k*_1_.

The parameters describing the coordinate transformations are classified as extrinsic parameters and intrinsic parameters. The extrinsic parameters, which are the coefficients in the 4-by-4 matrix in Equation (1), describe how world coordinates transform to camera coordinates. The intrinsic parameters describe how camera coordinates transform (through normalized coordinates) to image coordinates. Based on the camera model used in this work (as shown in Equations (1) to (5)), the intrinsic parameters include focal lengths $f_{x}$ and $f_{y}$, principal points $c_{x}$ and $c_{y}$, and distortion coefficients $k_{1}$, $k_{2}$, $p_{1}$, $p_{2}$, $k_{3}$, $k_{4}$, $k_{5}$, and $k_{6}$. In practical applications, some of the distortion coefficients can be ignored (i.e., set to zeros) to simplify the solution of equations. Figure 2 shows an example image of a house with assumed camera parameters. The effects of selected intrinsic parameters can be seen by comparing the sub-figures in Figure 2. Smaller focal lengths reduce the sizes of objects and widen the angle of view (compare Figure 2a and Figure 2b). Principal points, if not in the center of an image, bias objects in the image (compare Figure 2a and Figure 2c). Figure 2d presents the distortion effect of *k*_1_. The effects of the $p_{1}$, $p_{2}$, $k_{2}$, etc. are similar to that of *k*_1_ but in higher order, and are not redundantly presented here.

## 3. Image Analysis Procedures

The image analysis method employed in this work comprises four main procedures: camera calibration, target tracking, synchronization, and stereo triangulation. They are introduced in the following sections.

### 3.1. Camera Calibration

Camera calibration is a procedure to estimate the intrinsic and extrinsic parameters of a camera. It is typically carried out by taking photos of a calibration object with features whose coordinates are known and analyzing the positions of these features in the image. For each known feature point, its world coordinates (i.e., (*x_w_*_,_ *y_w_*_,_ *z_w_*)^T^) and its image position (i.e., (*x_i_*, *y_i_*)^T^) are known. With a sufficient number of known feature points, the intrinsic and extrinsic parameters can be calculated by solving Equations (1)–(5). A chessboard is a widely used calibration object because it has multiple distinct black–white intersection corners and these are relatively easier to detect in the image. This work employs a computer vision library named OpenCV [30], which provides camera calibration subroutines that encapsulate these numerical strategies for solving equations of coordinate transformation. Based on given world coordinates and image coordinates of a sufficient number of known feature points, these functions are capable of calculating the intrinsic and extrinsic parameters.

**Figure 3:**
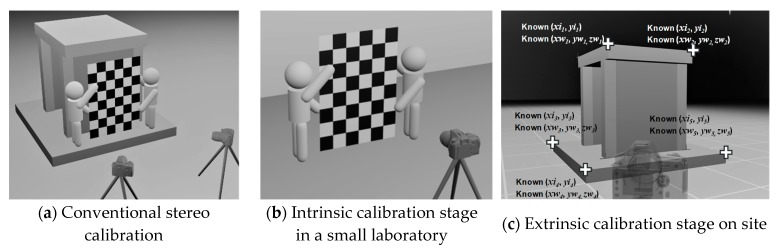
Different camera calibration approaches and stages. The proposed two-stage calibration includes intrinsic calibration in a small laboratory and extrinsic calibration on site.

Some studies have shown how stereo camera calibration can be carried out by taking photos of a calibration object using two cameras [29]. The aim of stereo camera calibration is to find out the intrinsic and extrinsic parameters of two cameras. Conventional stereo calibration is carried out by taking multiple pairs of photos of a calibration object positioned near the measurement region, with each pair of photos taken by both cameras simultaneously, as shown in Figure 3a. To achieve the best result, the calibration object needs to have a similar size to the measurement region and be positioned near the measurement region when taking the calibration photos. However, when the measurement region is very large, it becomes impractical or expensive to build a rigid calibration board large enough to reflect the cameras’ intrinsic parameters on site and in a structural laboratory.

In this research, two calibration approaches are introduced to estimate the camera parameters in large-scale experiments: (1) two-stage calibration and (2) single-image calibration. The two-stage calibration approach is suitable for cases where the measurement region is so large that it is impractical to build a similar-sized calibration board. In this approach, the intrinsic parameters and the extrinsic parameters are calibrated separately. Intrinsic parameters are calibrated in a relatively small laboratory by taking photos of a calibration board which does not need to be the same size of the large specimen (see Figure 3b). Without changing camera settings (e.g., focal lengths, focusing distance, etc.), re-positioning a camera does not change its intrinsic parameters. The extrinsic parameters obtained in the second stage, which is carried at the site of the large-scale experiment. With sufficient known calibration points, for which both their image coordinates and world coordinates are known (as shown in Figure 3c), a computer can solve the extrinsic parameters by numerically solving the nonlinear regression of Equations (1) to (5). It should be noted that although there are twelve extrinsic parameters shown in the 4 × 4 matrix in Equation (1), there are only six independent parameters (or degrees of freedom). Since the intrinsic parameters are already known, the solution of extrinsic parameters required fewer known points than intrinsic calibration does.

The single-image calibration approach is suitable for cases where only photos of the measurement region are available, and not those of calibration objects. This approach can be used for old experiments where camera calibration was not carried out and the cameras are not available for calibration now. In addition to the photos themselves, the only available information is the spatial information of the measurement region according to the design drawings of the specimen, so that the image coordinates of several known points in the measurement region, as well as their corresponding world coordinates, can be obtained. The single-image calibration is similar to the extrinsic calibration in the lab and on-site approaches (as shown in Figure 3c), except that the intrinsic parameters become unknowns and are to be solved together with the extrinsic parameters. Because of the increase in the number of unknowns, it is necessary to have relatively more known points to solve more unknowns or to reduce the number of intrinsic parameters by assuming some of the higher-order distortion coefficients (e.g., *p*_1_, *p*_2_, and *k*_2_ to *k*_6_) to be zeros, making it easier to converge when solving the numerical problem of coordinate transformation.

The calibrated intrinsic and extrinsic parameters need to be re-examined before performing any further image analysis to make sure these parameters do not converge to incorrect values. Since the calibration procedure involves solving nonlinear equations for coordinate transformation, improper selection of points could lead to incorrect results or an ill-posed system of equations. The following examinations are suggested:

1. Principal points (cx and cy): The principal points should be close to the center of an image. The center point of an image is at ((w−1)/2, (h−1)/2), assuming the image point of the upper-left pixel is (0, 0) (where w and h are the width and height of the image in terms of the number of pixels of images). For example, for a camera resolution of 3840 by 2160, the principal point should be close to (1919.5, 1079.5).
2. Focal lengths ($f_{x}$ and $f_{y}$): Focal lengths depend on the image resolution and the angles of view of the camera. For a typical full-frame single-lens reflex camera with a 55-mm lens, the focal lengths are approximately 1.5 times the width of the image. An 18-mm wide-angle lens would have a focal length approximately 0.5 times the width of the image, while a 105-mm narrow-angle lens would have one approximately three times the width. In addition, $f_{x}$ and $f_{y}$ should be approximately the same for most cameras.
3. Distortion coefficients: Larger magnitude of distortion coefficients induce more distortion effects in images. Even cameras and lenses with the same models and manufacturers may have slightly different distortion coefficients due to manufacturing imperfection. A wide-angle lens normally induces more distortion. Based on the author’s experiences, the values of coefficient $k_{1}$ of most cameras are normally between −0.5 and +0.5. Other coefficients (i.e., $p_{1},\;p_{2},\;k_{2},\;$…) are typically much smaller than 0.5. Distortion coefficients outside of these ranges should be double-checked.
4. Camera position: The camera position in the world coordinates can be calculated using the extrinsic parameters with:

(6) $$\left(\begin{array}{c} x_{p.camera}\\ y_{p.camera}\\ z_{p.camera} \end{array}\right)=-{\left(\begin{array}{ccc} R_{xx} & R_{xy} & R_{xz}\\ R_{yx} & R_{yy} & R_{yz}\\ R_{zx} & R_{zy} & R_{zz} \end{array}\right)}^{T}\left(\begin{array}{c} T_{x}\\ T_{y}\\ T_{z} \end{array}\right).$$

The camera’s viewing direction and its viewing axis (i.e., the z-axis camera coordinate) can be calculated as follows:

(7) $$\left(\begin{array}{c} x_{view.camera}\\ y_{view.camera}\\ z_{view.camera} \end{array}\right)={\left(\begin{array}{ccc} R_{xx} & R_{xy} & R_{xz}\\ R_{yx} & R_{yy} & R_{yz}\\ R_{zx} & R_{zy} & R_{zz} \end{array}\right)}^{T}\left(\begin{array}{c} 0\\ 0\\ 1 \end{array}\right)=\left(\begin{array}{c} R_{zx}\\ R_{zy}\\ R_{zz} \end{array}\right).$$

Since a user typically has a rough idea where the cameras were and in which directions they were shooting, the calculated camera positions and viewing axis should be checked if they match the actual installations in the laboratory. The 3-by-3 matrix in both equations is a normalized orthogonal matrix, so its transpose matrix is identical to its inverse matrix.

### 3.2. Target Tracking

**Figure 4:**
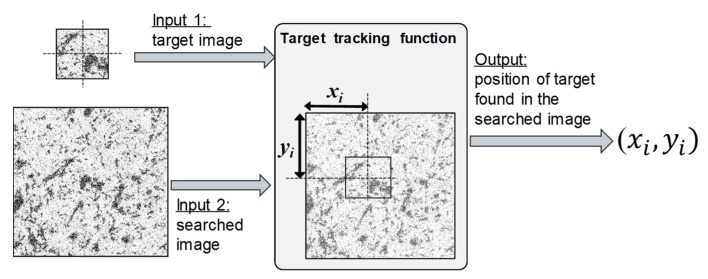
Input and output of a target-tracking function. Given target and searched images, a target tracking function returns the position where the target appears in the search image.

Target tracking finds the image position history of a certain point in a sequence of photos, that is, the time history of a certain point ($x_{i},\;y_{i})$. To measure the three-dimensional dynamic response of a target, it is necessary to repeatedly track its image position photo by photo in a video or photo sequence. Once the image coordinate ($x_{i},\;y_{i})$ of a certain target is obtained, its normalized coordinates ($x_{n},\;y_{n})$ can be calculated as Equations (1)–(5) are invertible. While a target is ideally a point with zero area, it is in practice represented by a small region of the image called a template. The template must contain sufficient unique image patterns. The efficacy of the target tracking methods depends on the contrast of the image patterns. In many applications a surface preparation, such as applying paint or spray that results in a random speckle pattern, is used to help target tracking algorithms. Quantitative error assessment has been done on the effects of image contrast and other factors [32]. Given the image of a target and a searched image that is supposed to contain the target, a target-tracking function finds the image position ($x_{i},\;y_{i})$ where the target is located in the search image (see Figure 4).

This work employs a multi-level template match method [13] to track the movement of points of interest. For each level of template matching, a trial image position is set and the template is compared with a same-sized cropped image around the trial image position in the searched image. If the trial image position is very close to the accurate position the target is, the two images would yield a large correlation coefficient (i.e., close to 1). Template matching tries all possible integer image positions (i.e., pixel by pixel) and picks the position that yields the largest correlation coefficient. Some researchers have implemented modified versions to speed up template-match computing [33]. To achieve a sub-pixel precision, after every level of template matching, both the template and searched images are enlarged for the next level of template matching, and the template matching operation is carried out again. However, the trial range is limited to near the image position calculated in the previous level. These operations can be iterated level by level to further achieve better precision. Early research shows that the precision can reach 0.04 pixels [13] or better if the image quality is good.

### 3.3. Synchronization

**Figure 5:**
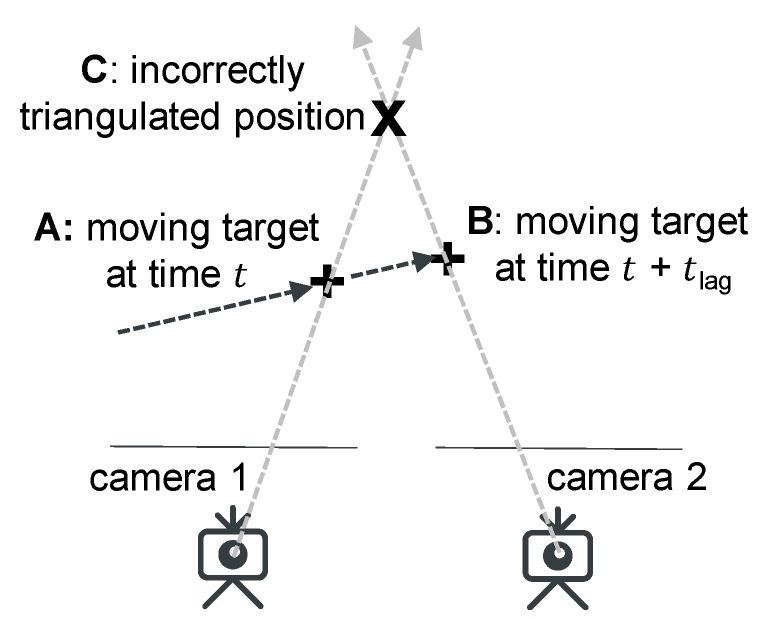
Incorrectly triangulated position caused by ignoring the time lag of camera 2. Even a small time difference between cameras could cause significant unexpected measurement error.

Synchronization between two cameras needs to be carried out before performing stereo triangulation, because it assumes both photos are taken simultaneously, so that objects in both photos have the same world coordinates. Integrating signals from different systems of sensors encounters synchronization issues [34]. However, even if both cameras were triggered at nearly the same time, a time difference of tens of milliseconds can lead to significant errors. As shown in Figure 5, the target moves from point A to B during the time lag, leading to an incorrectly triangulated point C, which is possibly not even close to the moving path of the target.

In this work, cross correlation is employed to estimate the time difference between cameras. Cross correlation is a signal-processing approach to measure the time lag and similarity between time series ${\bar{v}}_{t}^{L}$ and ${\bar{v}}_{t}^{R}:$

(8) $${\bar{v}}_{t}^{L}=\;(v_{t}^{L}-\mu^{L})/\sqrt{\underset{i}{\sum }{\left(v_{i}^{L}-\mu^{L}\right)}^{2}}$$

(9) $${\bar{v}}_{t}^{R}=\;(v_{t}^{R}-\mu^{R})/\sqrt{\underset{i}{\sum }{\left(v_{i}^{R}-\mu^{R}\right)}^{2}}$$

where $v_{t}^{L}$ and $v_{t}^{R}$ are the measured moving velocity (pixels per frames) of a certain point at time *t* in two image coordinates of the left and right images, respectively. The $\mu^{L}$ and $\mu^{R}$ are the mean values of movement increment in a certain time period. The ${\bar{v}}_{t}^{L}$ and ${\bar{v}}_{t}^{R}$ are the normalized and dimensionless time series of $v_{t}^{L}$ and $v_{t}^{R}$. Each correlation operation is given a correlation coefficient of ${\bar{v}}_{t}^{L}$ and ${\bar{v}}_{t}^{R}$, which indicates their similarity. By continuously shifting the time series with a time lag $t_{\mathrm{lag}}$, the similarity can be obtained as a function of $t_{\mathrm{lag}}$:

(10) $$C_{t_{\mathrm{lag}}}=\left({\bar{v}}_{t}^{L}\cdot {\bar{v}}_{t+t_{\mathrm{lag}}}^{R}\right),\;$$

and an optimized $t_{\mathrm{lag}}$ that results in the best similarity can be found. Assuming the target draws a similar trajectory on the image coordinates of both cameras, the $t_{\mathrm{lag}}$ that results in the best similarity of image movement histories of a certain target is an approximation of the time difference between the two cameras.

**Figure 6:**
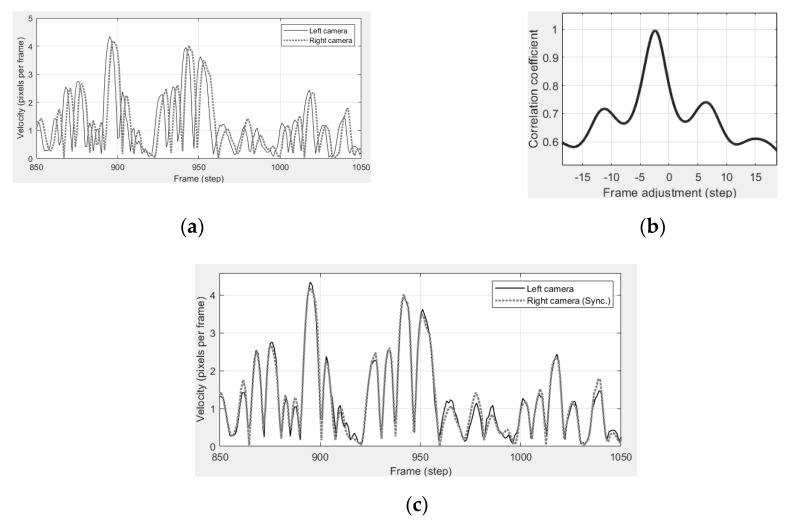
Synchronization by shifting image coordinate histories of the right camera. The proposed synchronization method estimates the time difference between cameras by applying cross correlation on analyzed moving velocity. (**a**) Non-synchronized movement. (**b**) Correlation vs. time lag. (**c**) Synchronized movement.

Figure 6a plots the moving velocity (pixels per frame) of a certain point taken by two cameras, showing a time lag between them. The velocity is calculated from the image coordinate history by using a finite-difference calculation. Figure 6b corrects the time lag and shows the two curves fit well. The correlation versus time lag is shown in Figure 6c where the estimated time lag is at the peak of the correlation coefficient curve.

**Figure 7:**
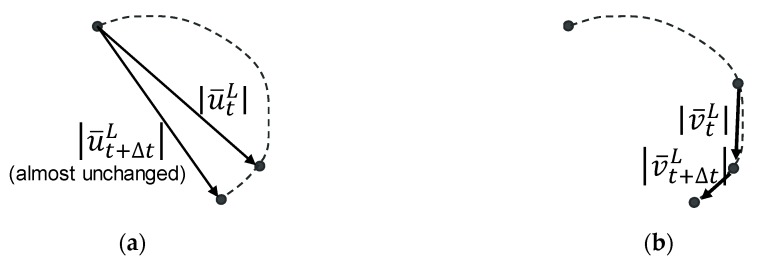
Displacement and velocity vectors of a moving point in image coordinate. The norm of the displacement vector does not represent the movement, making displacement an inappropriate indicator for synchronization. (**a**) Displacement vector history. (**b**) Velocity vector history.

The reason the velocity rather than the displacement is used for synchronization is that the velocity curves show better time lag consistency. The norm of the velocity vector represents the movement with reference to the previous time step, while the norm of the displacement vector represents the distance with the initial point. If the moving path of the selected point for synchronization is perpendicular to the displacement vector (as shown in Figure 7), the norm of the displacement vector (the distance to its initial position) is unchanged versus time, making the norm of displacement an inappropriate indicator for synchronization. The norm of vector is used rather than using x and y components separately for synchronization because the left and right cameras have different camera orientations (sometimes even almost 90 degrees different) and the movement along x (or y) in image coordinate could by quite irrelevant between two cameras.

**Figure 8:**
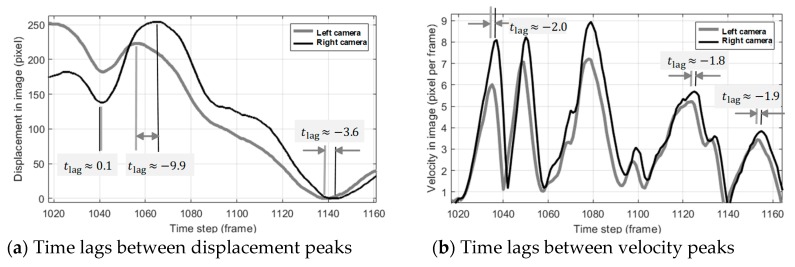
Comparison of synchronizations by matching moving velocity and absolute displacement. Synchronization based on velocity results in a consistency result.

Figure 8a shows the displacement and velocity of a certain point in images taken by two cameras in an experiment. It shows that the time difference between two curves apparently varies with time (with an inconsistency from 0.1 to −9.9 frames, up to 10 frames inconsistency), while the time difference between velocities (see Figure 8b) is more consistent (between −1.8 and −2.0 frames). The inconsistency of time lags estimated by using the norm of displacement vector may come from two sources: (1) taking the norm value of a vector, which eliminates property of orientation changes, and (2) dependency on the initial positions, which is inconsistent between two cameras. The revision is made as follows. In this point of view, increasing the sampling rate of cameras does not seem to solve the synchronization issue in this case.

### 3.4. Triangulation

**Figure 9:**
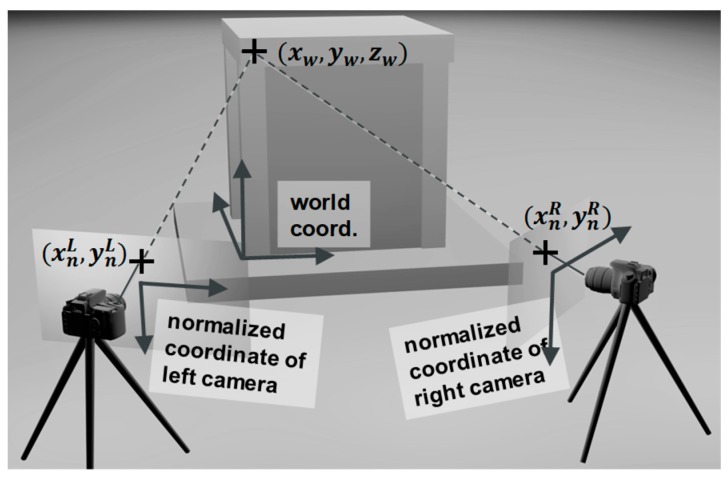
Three-dimensional positioning by using stereo triangulation. Two cameras are needed to position arbitrary point in three dimension.

Triangulation is widely used to obtain the three-dimensional world coordinates of a point. According to Equations (1) to (5), once the intrinsic and extrinsic parameters are known after camera calibration, any arbitrary point can be projected from its world coordinates ($x_{w}$ $y_{w}$, $z_{w}$) to its image coordinates ($x_{i}$, $y_{i}$). However, theoretically, a single camera cannot analyze the three-dimensional world coordinates of a certain point in the image mainly because its $z_{c}$ is unknown, making Equation (2) not invertible. Geometrically, two cameras are needed to position a point, as shown in Figure 9.

Given the image coordinates of a certain point in the left and right cameras $\left(x_{i}^{L},y_{i}^{L}\right)$ and $\left(x_{i}^{R},y_{i}^{R}\right)$, respectively, their projection points on normalized coordinates $\left(x_{n}^{L},y_{n}^{L}\right)$ and $\left(x_{n}^{R},y_{n}^{R}\right)$ can be calculated using Equations (1) to (5). The following equations help to solve the depths of both cameras, $z_{c}^{L}$ and $z_{c}^{R}$:

(11) $$\left(\begin{array}{c} \begin{array}{c} x_{n}^{L}z_{c}^{L}\\ y_{n}^{L}z_{c}^{L}\\ z_{c}^{L} \end{array}\\ 1 \end{array}\right)=\left(\begin{array}{cc} \begin{array}{ccc} R_{xx}^{L} & R_{xy}^{L} & R_{xz}^{L}\\ R_{yx}^{L} & R_{yx}^{L} & R_{yz}^{L}\\ R_{zx}^{L} & R_{zy}^{L} & R_{zz}^{L} \end{array} & \begin{array}{c} T_{x}^{L}\\ T_{y}^{L}\\ T_{z}^{L} \end{array}\\ \begin{array}{ccc} 0\;\;\;\; & 0\;\;\;\; & 0\;\; \end{array} & 1 \end{array}\right)\left(\begin{array}{c} \begin{array}{c} x_{w}\\ y_{w}\\ z_{w} \end{array}\\ 1 \end{array}\right)\;$$

(12) $$\left(\begin{array}{c} \begin{array}{c} x_{n}^{R}z_{c}^{R}\\ y_{n}^{R}z_{c}^{R}\\ z_{c}^{R} \end{array}\\ 1 \end{array}\right)=\left(\begin{array}{cc} \begin{array}{ccc} R_{xx}^{R} & R_{xy}^{R} & R_{xz}^{R}\\ R_{yx}^{R} & R_{yx}^{R} & R_{yz}^{R}\\ R_{zx}^{R} & R_{zy}^{R} & R_{zz}^{R} \end{array} & \begin{array}{c} T_{x}^{R}\\ T_{y}^{R}\\ T_{z}^{R} \end{array}\\ \begin{array}{ccc} 0\;\;\;\; & 0\;\;\;\; & 0\;\; \end{array} & 1 \end{array}\right)\left(\begin{array}{c} \begin{array}{c} x_{w}\\ y_{w}\\ z_{w} \end{array}\\ 1 \end{array}\right)\;$$

Since the world coordinates $\left(x_{w},\;y_{w},\;z_{w}\right)$ in Equations (11) and (12) are supposed to be the same in both cameras, the two equations can be reorganized and simplified to:

(13) $$A\left(\begin{array}{c} z_{c}^{L}\\ z_{c}^{R} \end{array}\right)=B$$

where A is a 3-by-2 matrix and B is a 3-by-1 matrix, respectively. Both of the left and right parts of the matrix A are calculated by multiplying a 3-by-3 matrix and a 3-by-1 vector, as shown in Equation (14). Equation (15) illustrates how the 3-by-1 matrix B is calculated:

(14) $$A=\left(\begin{array}{cc} {\left(\begin{array}{ccc} R_{xx}^{L} & R_{xy}^{L} & R_{xz}^{L}\\ R_{yx}^{L} & R_{yx}^{L} & R_{yz}^{L}\\ R_{zx}^{L} & R_{zy}^{L} & R_{zz}^{L} \end{array}\right)}^{T}\left(\begin{array}{c} x_{n}^{L}\\ y_{n}^{L}\\ 1 \end{array}\right)\;\; & \;\;\;\;\;\;\;{\left(\begin{array}{ccc} R_{xx}^{R} & R_{xy}^{R} & R_{xz}^{R}\\ R_{yx}^{R} & R_{yx}^{R} & R_{yz}^{R}\\ R_{zx}^{R} & R_{zy}^{R} & R_{zz}^{R} \end{array}\right)}^{T}\left(\begin{array}{c} x_{n}^{R}\\ y_{n}^{R}\\ 1 \end{array}\right) \end{array}\right)$$

(15) $$B={\left(\begin{array}{ccc} R_{xx}^{L} & R_{xy}^{L} & R_{xz}^{L}\\ R_{yx}^{L} & R_{yx}^{L} & R_{yz}^{L}\\ R_{zx}^{L} & R_{zy}^{L} & R_{zz}^{L} \end{array}\right)}^{T}\left(\begin{array}{c} T_{x}^{L}\\ T_{y}^{L}\\ T_{z}^{L} \end{array}\right)-{\left(\begin{array}{ccc} R_{xx}^{R} & R_{xy}^{R} & R_{xz}^{R}\\ R_{yx}^{R} & R_{yx}^{R} & R_{yz}^{R}\\ R_{zx}^{R} & R_{zy}^{R} & R_{zz}^{R} \end{array}\right)}^{T}\left(\begin{array}{c} T_{x}^{R}\\ T_{y}^{R}\\ T_{z}^{R} \end{array}\right)$$

Since the 3-by-3 matrices in Equations (14) and (15) are normalized orthogonal matrices, their inverses are equal to their transposes. The matrices A and B can be calculated by using the known extrinsic parameters of the cameras. Equation (13) contains two unknowns and three linear equations. By using the least squares method, it can be solved by:

(16) $$\left(\begin{array}{c} z_{c}^{L}\\ z_{c}^{R} \end{array}\right)={\left(A^{T}A\right)}^{-1}A^{T}\;B$$

Once $z_{c}^{L}$ and $z_{c}^{R}$ are obtained, the world coordinates $\left(x_{w},\;y_{w},\;z_{w}\right)$ can be calculated by using Equations (11) and (12).

While two cameras are mathematically sufficient to position a point in a 3D space, more than two cameras are sometimes adopted. They can be used in the following situations:

5. More than two cameras give spare cameras in case that any camera fails to capture clear videos.
6. If the movement of a tracking point is so large that it can run out of the field of view of any camera, it may require more cameras so that the point can be captured by at least two cameras at all time.

Since the calibration method proposed in this paper is applied to cameras one by one separately, as shown in Figure 3b,c, camera calibration for more than two cameras can be applied in the same manner. In addition, the OpenCV package also briefly introduces how to carry out multi-camera calibration in its documentation [30].

## 4. Experiments

In this section, the measurement of the three-dimensional displacement history of dynamic experiments using the image analysis method is demonstrated. Both experiments were shake table experiments conducted using two consumer video recorders.

### 4.1. Table Motion and Story Drift of 3-Story RC Experiment

The experiment involves a three-story reinforced-concrete (RC) building specimen with one span in the *x* direction (north–south) and two spans in the *y* direction (east–west). The second and third floors have shear walls on the south side. The shear walls are painted in white without additional painted image pattern, thus, are not measured by image analysis. The first story is entirely open without walls. The building is subjected to a uniaxial near-field ground motion. The ground motion used in this experiment was chosen to have a near-fault effect on structures, in order to validate and demonstrate the capability of a new shake table designed for near-fault ground motions that was recently constructed in the laboratory. A near-fault ground motion typically induces relatively high velocity peaks and large displacements, making it difficult to reproduce on a shake table, owing to the hardware limitations on actuator strokes. In this experiment, the demand ground motion is based on actual accelerations recorded by a station near an active fault in the magnitude-7.6 Chi-Chi earthquake in 1999. The largest displacement reaches 1.9 m, while the shake table has a maximum displacement of 2 m.

The global displacement of this experiment is difficult to measure with conventional sensors, because of its large size. It is impractical to build a large reference frame with sufficient rigidity for fixity of sensors aside the specimen. Three displacement measurement systems were employed in this experiment: (1) linear variable differential transducers (LVDTs) with a nominal accuracy of 0.05 mm at a sampling rate of 200 Hz to measure the relative displacements between stories, (2) an industrial infrared vision based remote sensing system that track passively reflective markers attached on the beam-column joints with a nominal accuracy of 1 to 0.1 mm (depending on spatial configuration and many factors) at a sampling rate of 180 Hz, and (3) consumer video cameras which take videos with 3840 by 2160 resolution at a 29.97-frame-per-second frame rate, and are analyzed by the image measurement method developed in this work.

**Figure 10:**
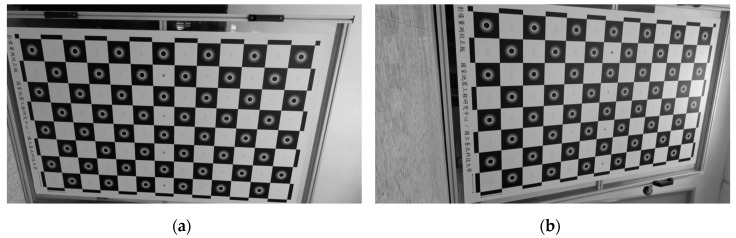
Selected samples of video snapshots for intrinsic calibration. The calibration photos are used for the first stage of the two-stage calibration approach. (**a**) Video snapshot of the left camera. (**b**) Video snapshot of the right camera.

The two-stage approach is adopted for image analysis in this experiment. Conventional stereo calibration, which is the method mentioned in Section 2, is not practical in this experiment, because it is difficult to build a 6-m-high rigid calibration board. The intrinsic parameters were calibrated in the laboratory using a roughly A0-sized calibration board. The intrinsic parameters of each camera (i.e., left and right) were calibrated according to video clips taken indoor, as shown in Figure 10.

Table 1 lists the calibrated intrinsic parameters. The aspect ratios (i.e., $f_{x}$ over $f_{y}$) of two cameras are 1.001 and 1.002, respectively. The principal points $c_{x}$ and $c_{y}\;$are less than 60 pixels away from the center of the image (whose width and height are 3840 and 2160 pixels, respectively). The higher-order coefficients $k_{3}$ to $k_{6}$ were assumed to be zeros. In the author’s experience, most of the cameras have an aspect ratio (i.e., $f_{x}$ over $f_{y}$) between 0.98 and 1.02, and the distance between the principal point and the center of the photo is normally less than 20% of the diagonal of the entire photo. While reasonable ranges of the aforementioned camera parameters depend on many factors and researchers many have subjective criteria, some studies investigated some of the parameters and their possible impacts to measurement errors [35,36]. If the focal lengths and the principal point are not within in these ranges, it should be checked that if the calibration photos are taken clearly, corners in the chessboard are correctly captured or not. If necessary, intrinsic parameters could need to be re-calibrated again.

**Figure 11:**
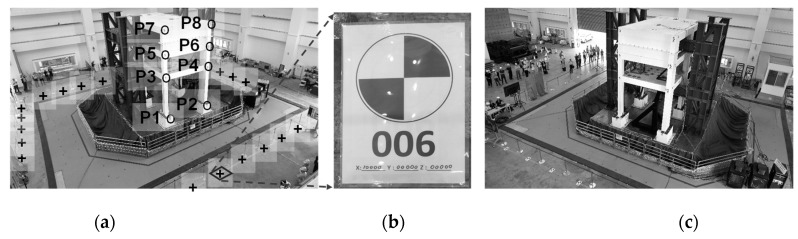
The 3-story RC building specimen and known calibration points marked. Points data in these photos are used for the second stage of the two-stage calibration approach. (**a**) Left camera view. (**b**) Physically deployed marker. (**c**) Right camera view.

**Figure 12:**
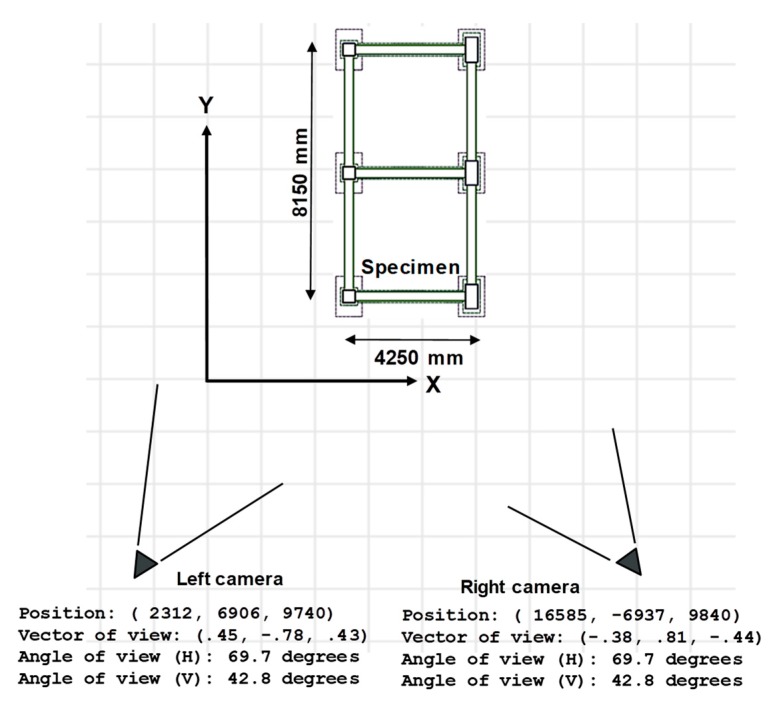
Camera positions and viewing directions based on calibrated extrinsic parameters. Calibrated extrinsic parameters represent the positions and orientations of cameras in the experiment.

The extrinsic parameters were calibrated by using some known calibration points surrounding the shake table, as shown in Figure 11. The calibration points were black–white markers, as shown in Figure 11b. The crosses in Figure 11a indicate the positions of known calibration points. The extrinsic parameters (i.e., $R_{xx},\;\ldots ,\;R_{zz},\;T_{x},\;T_{y},\;T_{z})$ can be estimated by solving Equations (1) to (5) with the given intrinsic parameters shown in Table 1. The positions and viewing directions of both cameras can be estimated by using the calibrated extrinsic parameters and are depicted in Figure 12. The position and viewing direction of cameras should be double-checked whether they match the actual installations on site or not. To our knowledge, there are no previous studies that can objectively give specific ranges of errors of extrinsic parameters. Some literatures discussed the sensitivity of extrinsic parameters and their possible impacts to the measurement accuracy [30,37]. In the author’s experience, the extrinsic parameters could be unreasonably wrong (e.g., the calculated camera position is hundreds of meters away from the laboratory, or at the opposite side of the ground) if the calibration points lead to an ill-conditioned problem. It normally occurs if the calibration points are nearly distributed along a line in the photo. If so, more calibration points are needed and the extrinsic calibration needs to be redone again.

**Figure 13:**
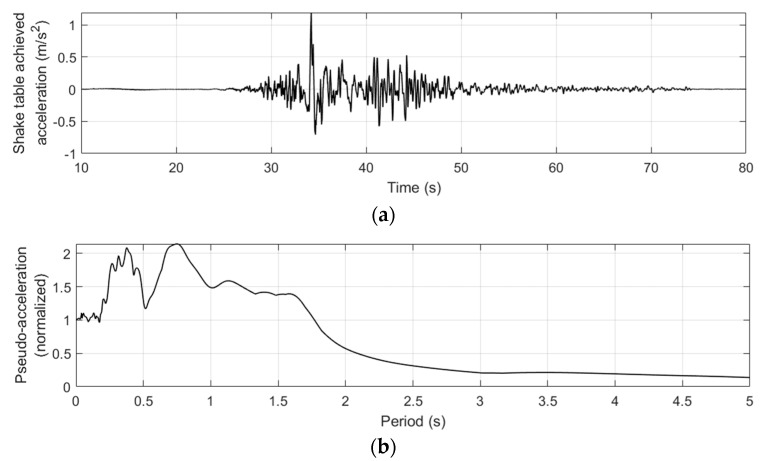
Ground acceleration history and response spectrum of the achieved shake table motion. (**a**) Time history of table acceleration along horizontal direction. (**b**) Response spectrum of the table acceleration along horizontal direction.

The ground motion inputted in the shake table control system consists of a biaxial acceleration history. The acceleration history is based on an actual acceleration record of one of the ground motion statins in the 1999 ChiChi earthquake in Taiwan, and is then adjusted to match the limited acceleration and stroke capacities of the shake table system. The adjustment of the ground motion is processed in both frequency domain and time domain through a sophisticated process, and is not described in this paper. The actually achieved horizontal acceleration in this experiment is shown in Figure 13a and its pseudo-acceleration response spectrum is plotted in Figure 13b.

**Figure 14:**
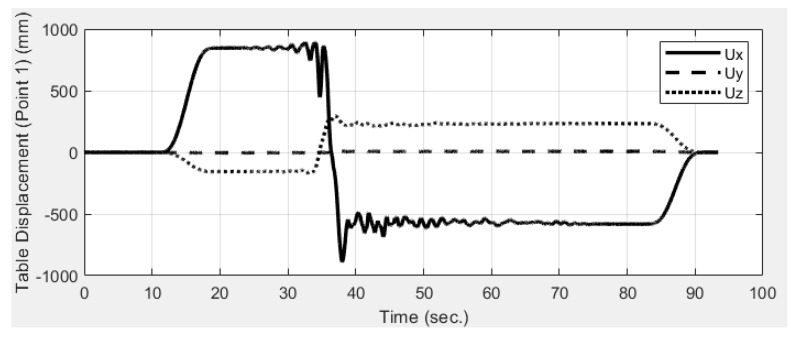
Table movement measured by image analysis. Shake table movement was estimated the image analysis.

Eight points marked as P1 to P8 in Figure 11a were measured via image tracking, synchronization, and stereo triangulation based on the calibration parameters. Markers of P1 to P8 are black-white squares as shown in Figure 11b. The high contrast of the markers aims to improve the efficacy of target tracking. The table movement was estimated by tracking the displacement histories of Point 1 (see Figure 14). The table moved to the right by 846 mm before a near-fault ground motion started. After the ground motion, the table moved back to the initial position. The table motion histories along the horizontal (*x*) and vertical (*z*) directions are as expected, and the out-of-plane movement (*y*), which was supposed to be zero, remained small (between −6 and 10 mm) according to the image analysis results. 

**Figure 15:**
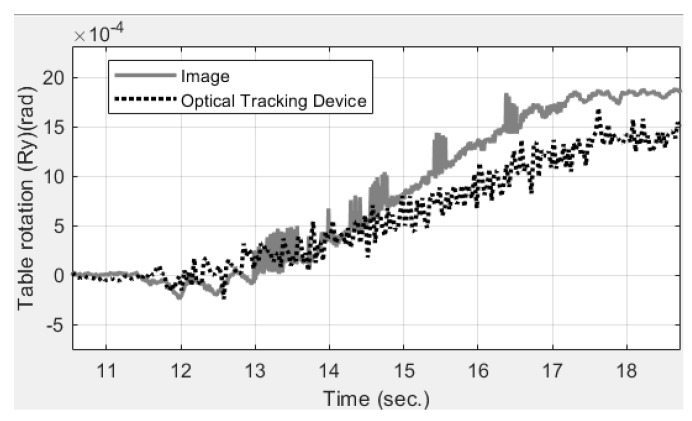
Table rotation (along the *y* axis) estimated by image analysis and an optical tracking device. Image analysis measures table rotation, which is difficult to measure by conventional sensors.

In addition to the displacement, the table rotation history was also estimated by analyzing the vertical (*z*) displacement histories of points 1 and 2. The image analysis shows that the table rotated by 0.0018 rad (i.e., approximately 0.1°) when moving to the right-hand side before near-fault ground motion was inputted. The table motion was measured by an optical tracking device, which indicates the rotation was 0.0015 rad (i.e., 0.086°), as shown in Figure 15. While the rotation seems small, the effect of rotational error on measuring the horizontal displacement can be amplified by the structural height. For a 6.73-m-high structure table, a small table rotation of 0.0018 rad could have induced more than 12 mm of horizontal drift and unexpected P-Delta effects on columns. However, this rotation was not able to be measured by other local sensors instrumented inside the RC structure.

Due to the slight table rotation, the story drift of the first floor cannot be estimated by simply calculating the differences in horizontal position between points 1 and 3. In this work, the story drift history of the first floor is estimated by calculating the angular variation multiplied by the column height, as given below:

(17) $$v_{1}=P_{1}-P_{3},$$

(18) $$v_{2}=P_{4}-P_{3},$$

(19) $$\theta =cos^{-1}\left(\frac{v_{1}\cdot v_{2}}{\left|v_{1}\right|\left|v_{2}\right|}\right),\;\mathrm{and}$$

(20) $$u_{1}=\left|v_{1}\right|\left(\theta -\theta_{0}\right).$$

**Figure 16:**
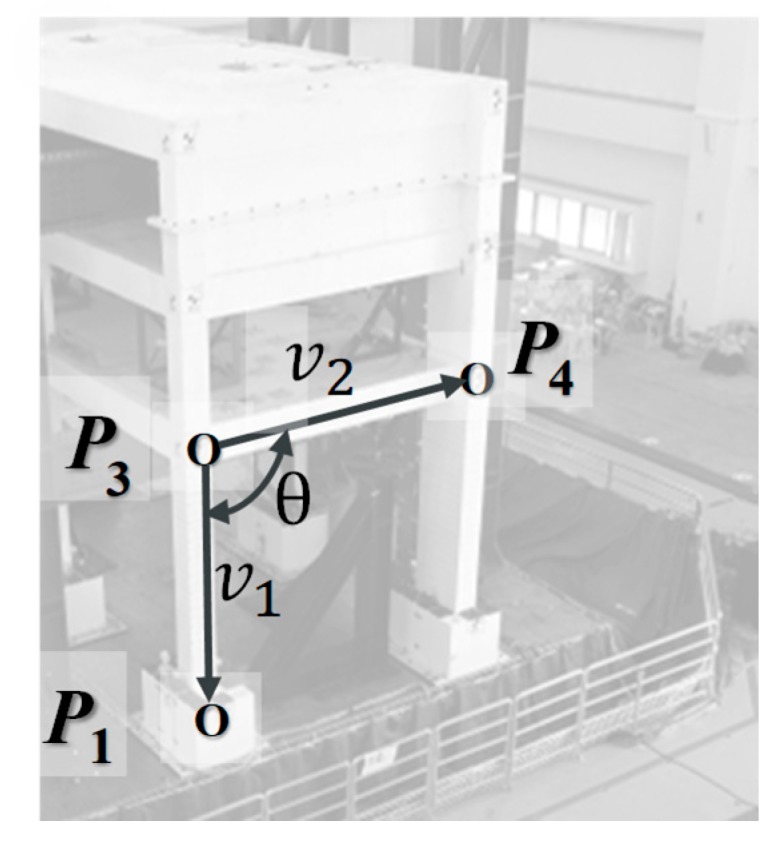
Estimation of first-floor drift angle. The story drift history is estimated by angle calculation to avoid error induced by the shake table rotation.

Once the world coordinates of points 1, 3, and 4 are calculated by triangulation, the vectors $v_{1}$ and $v_{2}$ and the angle θ can be calculated (see Figure 16). The estimated first-floor drift displacement $u_{1}$ was then estimated by the change in the angle with respect to the initial angle $\theta_{0}$ multiplied by the estimated column height. 

**Figure 17:**
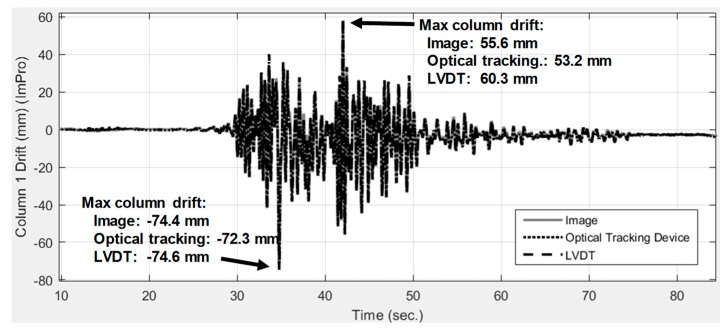
Comparison of three different measurement system results for first-story drift history. Image analysis gives satisfactory accuracy comparing with other sensors.

The image measurement results were used to estimate the time history of the story drift of the first floor and were compared with the optical tracking device and LVDT displacement sensors. The first-floor drift was estimated using the time history of the world coordinates of points 1, 3, and 4 analyzed from the images. These measurement approaches are all capable of capturing the general response of the story-drift history. One of the largest differences is at the response peak around 42 s, which reached a difference of 4.7 mm between the image analysis (55.6 mm) and LVDT results (60.3 mm), as shown in Figure 17.

**Figure 18:**
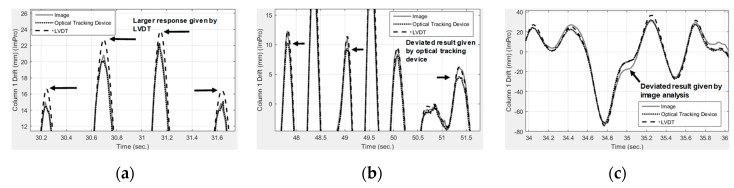
Responses where one device deviated from the others. The image analysis reaches the same accuracy when comparing with the optical tracking and LVDT devices. (**a**) Peaks where LVDT results were higher than others. (**b**) Peaks where optical tracking results were lower than others. (**c**) Duration where image analysis were different from others.

Most of the differences between the image analysis and LVDT results at the response peaks were within 2 mm. Image analysis, the optical tracking device, and the LVDT produced generally similar response results (see Figure 18). The LVDT generally produced 1-mm to 3-mm higher responses at the drift ratio peaks (see Figure 18a). The maximum measurement difference between two industrial-level measurement devices (i.e., the optical tracking device and the LVDT) at the displacement peaks is 3 mm. The maximum measurement difference between image analysis and the industrial-level measurement is less than 3 mm at the displacement peaks, indicating that the image analysis based on consumer products with proper synchronization has potential to reach the same accuracy with industrial-level measurement. It is also observed that image analysis produced an error as large as 10 mm, as shown in Figure 18c, probably because of synchronization errors between the two cameras. Considering that the absolute displacement of the first floor is 893 mm, the maximum relative error of image analysis is 0.33% (i.e., 3/893) at peaks and is 1.11% (i.e., 10/893) at non-peak regions. While LVDT and optical tracking device exist a 0.33% difference with each other, the image analysis reached the same accuracy at peaks. These slight differences also reflect the needs of using more than one type of measurement devices on important measurement or structural monitoring applications, as measurement methods have different source of errors, especially under environments with much random noise, strong vibrations, and possible mounting failure of markers or devices.

**Figure 19:**
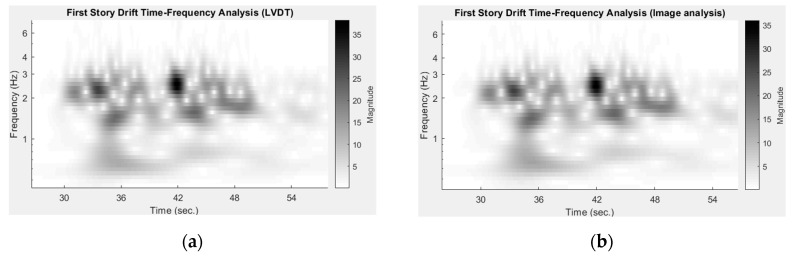
Time–frequency results of using the LVDT and image analysis. Time–frequency verifies the accuracy of image analysis. (**a**) Using the LVDT. (**b**) Using image analysis.

Comparing the time–frequency results of the LVDT and image analysis, the differences between them are less than 2 mm in the time-frequency intensity shown in Figure 19.

**Figure 20:**
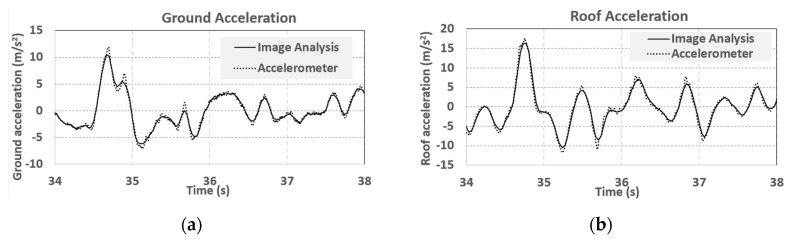
Acceleration histories from accelerometers and image analysis. High–frequency noise could be generated by estimating acceleration based on image measurement. (**a**) Ground acceleration. (**b**) Roof acceleration.

The time–frequency results are produced by a continuous wavelet transform [38]. It can be seen that the image analysis of three-dimensional point tracking using consumer video cameras is capable of capturing the overall dynamic responses of structural experiments. However, it should be noted that the sampling rate of image analysis (i.e., 29.97 frames per second) is much lower than that of the LVDT (i.e., 200 Hz), and the results would be worse at higher frequencies. High speed cameras should be used for image analysis when high frequency responses of displacement measurements are required. The acceleration responses of structures can be estimated from the second derivative of the displacements [39]. While image analysis can be used to estimate the positions of objects, it does not naturally detect any information about acceleration. This work estimates acceleration histories by using a central difference method [31] with a low-pass filter dropping out frequencies higher than 15 Hz (about a half of the video frame rate). The ground acceleration and roof acceleration histories are estimated and compared with the results of an accelerometer installed on the specimen, as shown in Figure 20a,b. The sampling rate of the accelerometers was 200 Hz, which is approximately five times higher than that of the video cameras (29.97 frames per second). The maximum ground acceleration measured by the accelerometer is 11.91 m/s^2^, but is 10.50 m/s^2^ measured by image analysis, as shown in Figure 20a. The differences of acceleration histories obtained by image analysis and the accelerometers are less than 1 m/s^2^ at non-peak regions but reach up to 1.41 m/s^2^ at acceleration peaks, which is equivalent to a relative error of 11.8% (i.e., 1.41/11.91). The image analysis also includes relatively larger high-frequency noise before using the aforementioned low-pass filter. The high-frequency noise is induced by error propagation effects and can be reduced with signal processing techniques [39].

### 4.2. Brick-Walled RC Frame Experiment

A brick-walled one-story RC frame was tested on a shake table in 2006 [40]. Two of the videos taken in this experiment were used in this present work to demonstrate how to perform image analysis on relatively old videos or photos where calibration was not carried out during the experiment. The displacement sensors were very limited, owing to the high risk of damage to these expensive sensors by falling bricks. An LVDT sensor was used in the experiment that measured the horizontal displacement of the shake table with a nominal accuracy of 0.1 mm at a sampling rate of 200 Hz. Due to the lack of quantified data, the dynamic responses of brick wall deformation could only be observed through visual examination. Two videos taken during the experiment were adopted to estimate the deformation of the brick walls. The resolution of the video was 1440 by 1080 at a nominal 29.97-frame-per-second frame rate. However, no calibration was carried out during the experiment and the video camera hardware used are no longer available for carrying out intrinsic calibration.

**Figure 21:**
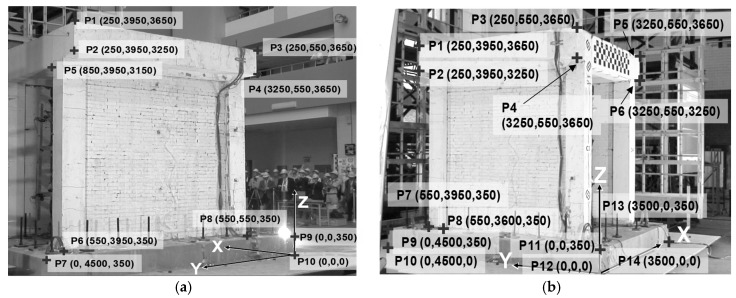
Snapshots of the two cameras and selected calibration points. (**a**) Left camera. (**b**) Right camera.

Without sufficient chessboard calibration photos of this experiment, the single-image calibration approach (i.e., intrinsic-and-extrinsic by user points on site) is employed to estimate the intrinsic and extrinsic parameters of the cameras. The user points were selected from some feature points that were clearly recognizable in all frames of the videos and for which world coordinates could be determined from the design drawings [40]. Figure 21 presents the initial snapshots of the two videos and the selected calibration points.

The intrinsic parameters and the extrinsic parameters were obtained by using the single-image approach based on the world coordinate and image coordinates of the calibration points shown in Figure 21. The estimated intrinsic parameters were listed in Table 2, where $p_{1}$ and $\;p_{2}$ and $k_{2}$ to $k_{6}$ were assumed to be zeros to avoid spurious values induced by numerical instability because of the very limited number of calibration points.

**Figure 22:**
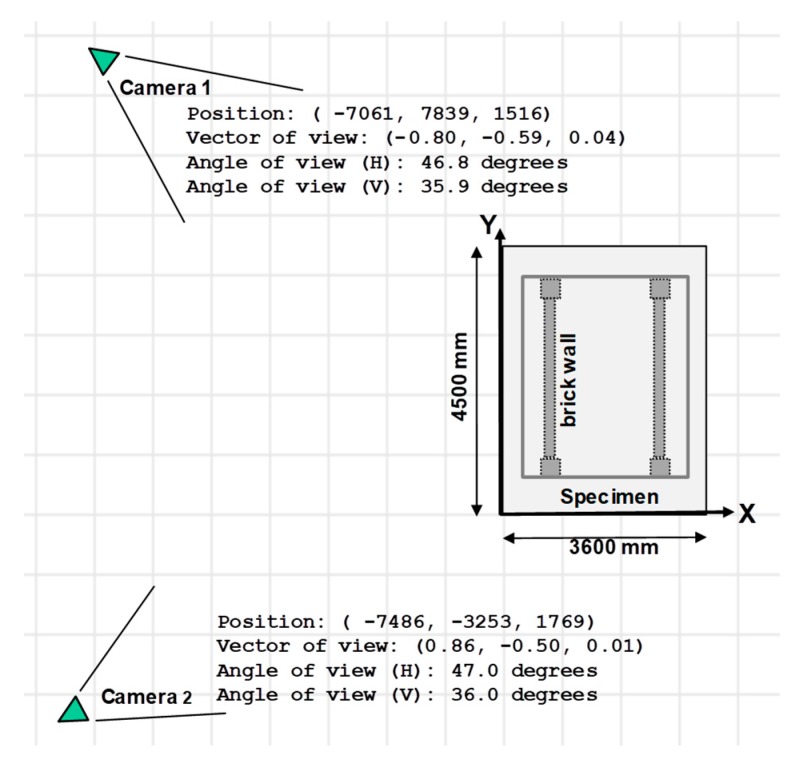
Estimated camera positions and viewing directions in the 1-story RC experiment. Calibrated extrinsic parameters represent the positions and orientations of cameras in the experiment.

It means only second-order radial distortion is considered. The aspect ratios (i.e., $f_{x}$ over $f_{y}$) of two cameras are 0.992 and 0.989, respectively. The principal points $c_{x}$ and $c_{y}$ are only 109 pixels away from the center of the photo (whose width and height are 1440 and 1080 pixels, respectively). The positions and viewing directions of both cameras can be estimated from the calibrated extrinsic parameters and are depicted in Figure 22.

**Figure 23:**
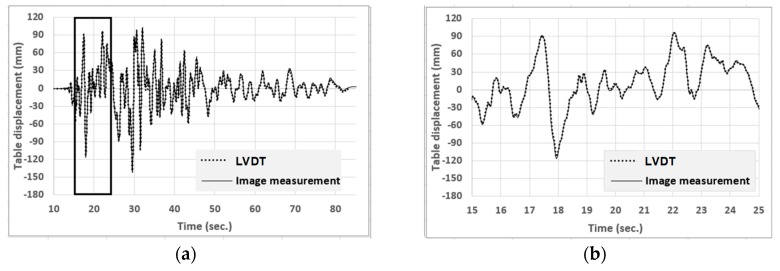
Table displacement histories from the LVDT and image analysis. Image measurement of table movement matches those of the LVDT. (**a**) Overall view (**b**) Magnification between 15 s and 25 s.

The displacement histories of the RC frame are estimated from image analysis using the calibration method, point-tracking method, synchronization, and triangulation. The table displacement histories measured from image analysis were compared with the results of the LVDT sensor, which was one of the few local sensors used in this experiment. The measured responses were similar (see Figure 23a), with differences in peaks of less than 2 mm (see Figure 23b) and the largest error not larger than 5 mm. While a 2-mm to 5-mm error might not be good enough for researches who require more precise measurement data, the image analysis measurement data presents the general structural responses, and is especially valuable for cases where local sensor data cannot be installed. Considering the video resolution was relatively low (i.e., 1440 pixels is width) and there was a lack of good calibration conditions in the old experiment, it is expected that image analysis accuracy would be much better for experiments carried out now.

**Figure 24:**
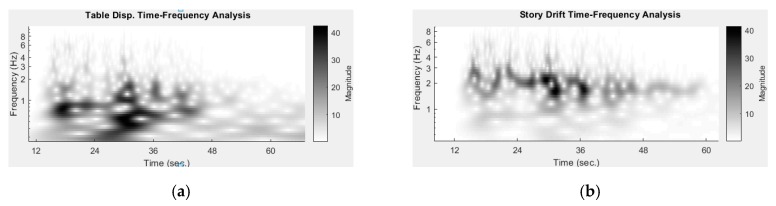
Time–frequency analysis of ground and roof displacements. Image analysis measurement shows the structural frequency variation, indicating a certain level of structural failure. (**a**) Foundation response(**b**) Roof response.

The ground and roof displacement histories from image analysis were subjected to a continuous wavelet transform to time–frequency data. Figure 24a shows that the frequency of ground motion was 0.7–1 Hz at 16 s and 17 s (named the first ground motion peak) and was 0–2 Hz as 28 s and 34 s (named the second ground motion peak).

**Figure 25:**
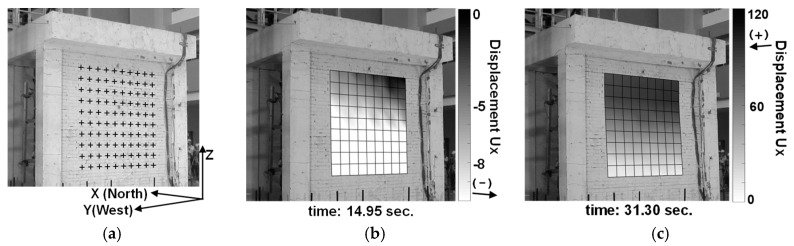
Wall torsion and bending observed from image analysis data. Image measurement quantifies structural dynamic responses that is difficult to acquire by local sensors in the experiment. (**a**) Mesh of tracked points. (**b**) Analyzed wall torsion (**c**) Analyzed wall bending.

Figure 24b shows that the first ground motion peak did not trigger a significant response of the roof, while the second peak was more influential. The response frequency of the roof appeared to be reduced after the second peak, from around nearly 3 Hz to around 2 Hz, indicating the structure was damaged to a certain degree by the second peak of the ground motion. In addition to measuring the displacements of predefined points, one of the best advantages of image analysis is being able to measure a large number of points distributed over a region. The dynamic deformation history of the whole brick wall in the shake table test could be estimated via image analysis. While no deliberate pattern was painted on the brick wall for image analysis in the old experiment, the dark edges between bricks can be used as a pattern required by image tracking. A mesh of tracking points (10 × 10) over the brick wall were chosen for image analysis, as shown in Figure 25a. However, due to the low contrast of the dark edges, the efficacy of the target tracking is limited. The displacement histories of these points were estimated through image point tracking, synchronization between two cameras, and stereo triangulation, thus providing the overall deformation history of the wall through the entire experiment. It can be visualized by plotting the displacement along the *x* axis on the wall, as shown in Figure 25b,c. Figure 25b presents a torsional deformation, which was caused by the separation between the wall and one of its adjacent columns [40]. While the torsion can be observed by watching videos, image analysis presents details and can quantify that the maximum torsion is 0.12° (i.e., an approximately 5-mm difference along the 2400-mm width). The plotted mesh in Figure 25c shows that bending deformation of the wall occurred at 31.2 s. While the structural behavior and failure mechanism are not the focus of this paper, the analyzed results demonstrate that image analysis is capable of quantifying some three-dimensional responses of some regions of interest for which conventional local sensors are not applicable.

## 5. Conclusions

In this work, an image analysis method and software implementation were developed to analyze the three-dimensional dynamic responses of shake table structural experiments. This method is especially practical in cases where conventional displacement measurement devices are difficult to apply. While three-dimensional image analysis requires the intrinsic and extrinsic parameters of the cameras used, this method is able to estimate these parameters even in older shake table tests where two videos were taken but their camera parameters are not available.

Two calibration approaches were introduced for large-scale experiments. The two-stage approach, which separates conventional stereo calibration into intrinsic calibration in a relatively small laboratory and extrinsic calibration on-site, is suitable in cases where the region of measurement is much larger than available calibration objects. This approach was employed in the shake table test of a three-story RC building, where the region of measurement was up to 6 m high, much larger than any calibration board available at that time. By using the two-stage approach, the intrinsic parameters were estimated by performing conventional intrinsic calibration using a relatively small calibration board, where the cameras’ configurations were set the same as they were in the shake table tests. The single-image approach, meanwhile, is suitable for older experiments where videos were taken but no calibration was performed and the cameras are no longer available for calibration. User points are selected where their image positions can be clearly recognized in the images and their world coordinates can be determined (normally based on specimen drawings). The single-image approach was adopted for analyzing videos taken in a 2006 shake table experiment. Camera parameters were estimated and three-dimensional image analysis was carried out.

In addition, a synchronization method was developed to minimize the stereo triangulation error induced by a time difference between two videos, as even a small difference can result in significant triangulation error. While manual video editing can roughly synchronize two videos, this method adopts cross-correlation analysis of the velocity histories from both videos for the same selected moving point. This method was verified through two experiments.

Two shake table experiments were used to demonstrate and verify the image analysis method for measuring dynamic displacement and calculating accelerations. They were compared with those obtained using a commercial optical motion-tracking system and conventional local sensors. It was also demonstrated that the measured points can be determined after the experiments (without the need to determine them beforehand) and can be a mesh of points that is distributed over a region and is dense enough to depict the dynamic responses of a region of interest.

## Figures and Tables

**Table 1:** Intrinsic parameters of cameras in the 3-story RC building experiment.

Parameter	Left Camera Intrinsic Parameters	Right Camera Intrinsic Parameters
$f_{x}$	2756.8	2763.3
$f_{y}$	2752.8	2756.1
$c_{x}$	1891.2	1908.2
$c_{y}$	1047.8	1065.6
$k_{1}$	0.01458	0.01964
$k_{2}$	−0.02843	−0.03392
$p_{1}$	−0.003156	−0.001522
$p_{2}$	−0.003009	−0.000871
$k_{3}$ to $k_{6}$	Set to zeros	Set to zeros

**Table 2:** Intrinsic parameters of the cameras in the 1-story brick-walled RC experiment.

Parameter	Left Camera Intrinsic Parameters	Right Camera Intrinsic Parameters
$f_{x}$	1663.9	1653.8
$f_{y}$	1676.1	1671.1
$c_{x}$	739.2	646.8
$c_{y}$	562.0	578.2
$k_{1}$	0.375	0.186
$p_{1}\;$and $p_{2}$ and $k_{2}$ to $k_{6}$	0	0
